# Clinical dermatoendocrinology: saving lives by looking at the skin

**DOI:** 10.20945/2359-4292-2025-0062

**Published:** 2025-11-24

**Authors:** Cynthia S. Barros-Oliveira, Carla R. P. Oliveira, Bruno de Santana Silva, Ângela C. Leal, Roberto Salvatori, Manuel H. Aguiar-Oliveira

**Affiliations:** 1 Divisão de Endocrinologia, Programa de Pós-graduação em Ciências da Saúde, Universidade Federal de Sergipe, Aracaju, SE, Brasil; 2 Divisão de Dermatologia, Departamento de Medicina, Universidade Federal de Sergipe, Aracaju, SE, Brasil; 3 Division of Endocrinology, Diabetes and Metabolism, Department of Medicine, The Johns Hopkins University School of Medicine, Baltimore, Maryland, United States

**Keywords:** Skin, endocrinology, dermatology

## Abstract

The separation of the interior from the exterior environment through the skin was fundamental for the evolutionary progression from prevertebrates into vertebrates. The development of the skin also established an internal environment controlled by hormones. The skin is influenced by different hormones; it is also the largest endocrine organ, producing several hormones. Skin inspections often save lives from skin cancer but can also diagnose potentially deadly endocrine diseases. The objectives of this review were to describe the emergence and definition of dermatoendocrinology and to focus on the clinical diagnosis of cutaneous manifestations of endocrine disorders, some of which are potentially fatal. This narrative review was based on a comprehensive search using the term “dermatoendocrinology” since its creation in 2001 in the PubMed^®^ database. Subsequently, a complementary search was performed with combinations of the keywords “skin”, “insulin”, “diabetes”, “thyroid”, “adrenal”, “sex hormones”, “parathyroid hormone”, and “growth hormone.” A total of 111 articles were included. The cutaneous manifestations of Itabaianinha syndrome (isolated growth hormone deficiency) and five anecdotal cases that enabled life-saving therapeutic measures are reported. The dermatoendocrine conditions described include acanthosis nigricans and androgenetic alopecia (insulin resistance), necrobiosis lipoidica diabeticorum and granuloma annulare (diabetes), pretibial myxedema (hyperthyroidism), xerosis cutis (hypothyroidism), purple striae and facial plethora (hypercortisolism), hyperpigmentation (primary adrenal insufficiency), dryness and urogenital atrophy (hypoestrogenism), hirsutism and virilization (hyperandrogenism), pruritus and calcium deposition (hyperparathyroidism), thinness, wrinkling, and reduced sweating (growth hormone deficiency), and thick oily skin with excessive sweating (acromegaly). Skin inspection allows the diagnosis of serious endocrinopathies.

## INTRODUCTION

In this review, we aim to reaffirm the currently overlooked concept that skin examination allows the diagnosis of endocrine diseases, whose treatment might be life-saving. We begin by describing the evolutionary relationship between skin and hormones, followed by a brief overview of the cutaneous anatomy and physiology, as well as the origins of dermatology, endocrinology, dermatoendocrinology, and neuroendocrinology of the skin. We conclude by developing the concept that endocrinologists, dermatologists, and general practitioners should routinely examine the skin, as it offers valuable insights into endocrine diagnostics relevant to their patients’ quality of life and lifespan.

The skin was the earliest organ to evolve, as evidenced by the existence of proto-skin in sponges (^1^). The separation between the internal and external environments provided by the skin was fundamental for the evolutionary progression from prevertebrates into vertebrates (^2^). In humans, the skin is composed of epidermis, dermis, and hypodermis. The epidermis is the outermost stratified layer, consisting of 95% squamous epithelial cells (keratinocytes) at various stages of differentiation (including the basal, spinous, granular, lucid, and corneal layers) in a cycle that lasts 2 to 4 weeks and concludes with cutaneous desquamation. Other cell types making up the epidermis include melanocytes, Langerhans cells (which serve immunological functions), and Merkel cells (which play a role in sensory perception). Melanocytes are derived from the neural crest and are distributed in the basal layer of the epidermis (one melanocyte is surrounded by approximately 36 keratinocytes), forming a complex network of interacting regulatory pathways that are essential for melanin’s protective effect against DNA-damaging ultraviolet (UV) rays (^3^). The basement membrane of the epidermis is attached to the dermis, which is rich in connective tissue with blood vessels, hair follicles, nerves, and immune cells. Structural extracellular matrix proteins - including collagen, elastin, various glycoproteins, and proteoglycans - provide the skin with tensile strength, resilience, and turgor and serve as a storage site for growth factors. Dermal connective tissue connects all cells and tissues of the skin as a functional organ (^4^). Fibroblasts are the main component of connective tissue; they produce and organize structural extracellular matrix proteins, interact with endogenous stem cell niches in the skin, and are essential for skin homeostasis and senescence (^4^,^5^). The subcutaneous fat tissue, which consists predominantly of adipocytes, plays a crucial role in regulating body temperature and providing mechanical protection to the inner organs. The evolution of bipedal posture may have driven the development of scalp hair to minimize heat gain from solar radiation, particularly in large-brained hominids. Hair undergoes a process consisting of three phases: the growth phase (anagen), lasting 2 to 6 years and comprising 90% of scalp hairs; the apoptosis phase (catagen), lasting 3 weeks and comprising less than 1% of scalp hairs; and the resting phase (telogen), comprising 10% of scalp hairs and not involving proliferation or apoptosis. Some authors also identify an additional phase, known as the exogen (or shedding) phase, during which the mature hair shaft is shed as new hair begins to grow (^6^). Another essential acquisition for thermoregulation is the activity of sweat glands. The eccrine glands open directly onto the skin surface and are distributed throughout the body. Apocrine glands are associated with hair follicles and are found in the armpits, anogenital region, eyelids, external auditory canals, and breasts. Sebaceous glands protect and moisturize the skin, hair, and other parts of the body through the production of sebum. They consist of the acinus and its sebocytes, which connect to the surface of the epidermis through an excretory duct that runs continuously with the hair follicle wall.

The evolution of skin established an internal environment controlled by secretions (homeostasis), which evolved into the concept of hormones. Although the origins of dermatology and endocrinology predate the birth of Jesus Christ, the scientific period for both specialties dates back to the 19th century. Scientific dermatology began in 1878 with the publication of the first section of the masterpiece On Cutaneous Diseases by Robert Willan (^7^). Endocrinology, in turn, emerged in the second half of the same century, building on the experimental studies conducted by Claude Bernard (1813-1878), the clinical observations of Thomas Addison (1793-1860), and the experimental and clinical studies of Brown-Séquard (1817-1894) (^8^).

Dermatologists and endocrinologists have long been fascinated by the complex relationship between hormones and human skin in health and disease (^8^). However, it was not until 2001 that dermatoendocrinology was established in Berlin with the founding of a working group by Christos Zouboulis, Ralf Paus, and Markus Böhm, within the *Arbeitsgemeinschaft Dermatologische Forschung* (^9^,^10^). This fundamental milestone established the concept that the skin is not only influenced by several hormones but is also the largest endocrine organ in the body, producing a series of hormones that have systemic or local action and express a myriad of cognate receptors, which are exposed to autocrine, paracrine, and intracrine mechanisms (^11^). Since then, dermatoendocrinology has been consolidated as the field that explores how hormones influence the skin and how skin conditions may reveal underlying endocrine disorders. It encompasses the research and clinical practice of understanding and managing skin conditions with an endocrine component, recognizing that the skin can reflect underlying hormonal imbalances (^12^). A current example can be found in aging research, in which the skin is used in models of mini-organs that not only respond to systemic hormones via cognate receptors but also synthesize and metabolize many of these hormones (^12^). In this sense, neurohormones produced within hair follicles on the human scalp exhibit mechanisms similar to those produced by the specialized hypothalamicpituitary-adrenal (^13^) and hypothalamic-pituitarysomatotropic axes (^14^). The study of these interactions is fundamental for understanding skin physiology and pathology, as well as for effectively treating dermatological conditions and delaying skin and overall aging through the development of new anti-aging therapies (^4^).

Currently, dermatoendocrinology and skin neuroendocrinology are venturing into new frontiers that neither conventional endocrine research nor investigative dermatology has explored extensively (^9^). These frontiers will be partially covered in the present article, which will describe the most relevant clinical interactions between the skin and the endocrine glands, with emphasis on the somatotropic system (^2^,^15^-^20^). This article is intended for general endocrinologists, dermatologists, and general practitioners and will not address the dermatological manifestations of rare hereditary endocrine syndromes, as it is not intended to be an exhaustive description of the cutaneous manifestations of endocrine diseases or to recommend endocrine and dermatological treatments for these conditions.

Skin inspection is often used to save lives from skin cancer (^21^,^22^), but it can also diagnose potentially deadly endocrine diseases, such as tanned skin suggesting the diagnosis of primary adrenal insufficiency.

## SUBJECTS AND METHODS

This narrative review summarizes quantitative studies without referencing the statistical significance of the results (^23^) and was conducted by one of the authors. based on a comprehensive search using the term “dermatoendocrinology” since its inception in 2001 in the PubMed^®^ database. A complementary search was subsequently performed using combinations of the keywords “skin”, “insulin”, “diabetes”, “thyroid”, “adrenal”, “sex hormones”, “parathyroid hormone”, and “growth hormone.” Additional articles were identified from the references cited in the retrieved articles. The inclusion criteria were peerreviewed articles published in English and Spanish. Exclusion criteria included articles with unavailable abstracts and conference abstracts. A total of 111 articles were retrieved, of which 13 were published by the present study’s authors. The final quality check of the literature selected for analysis was conducted by another authors.

This narrative review is complemented by our research experience with cutaneous findings in patients with Itabaianinha syndrome, characterized by isolated growth hormone (GH) deficiency (GHD) (^2^,^15^-^20^), along with nearly 25 years of clinical experience. Among the authors, the youngest clinician is an endocrinologist with 7 years of practice who holds both a master’s and a doctoral degree in the field, and the oldest clinician has 47 years of clinical practice. The authors also include a dermatologist, with 27 years of practice, who completed medical residency and holds a master’s degree in science. All authors except for one, work at the *Hospital Universitário de* Aracaju at the *Universidade Federeal de Sergipe*. From the patients receiving care in this institution, we selected anecdotal cases to demonstrate that a correct diagnosis enabled potentially life-saving therapeutic measures. The inclusion criteria were live patients with accurate clinical and biochemical diagnoses who were receiving appropriate therapeutic interventions (diet, medication, or surgery when indicated) and undergoing regular follow-up.

## RESULTS

**Figure 1:**
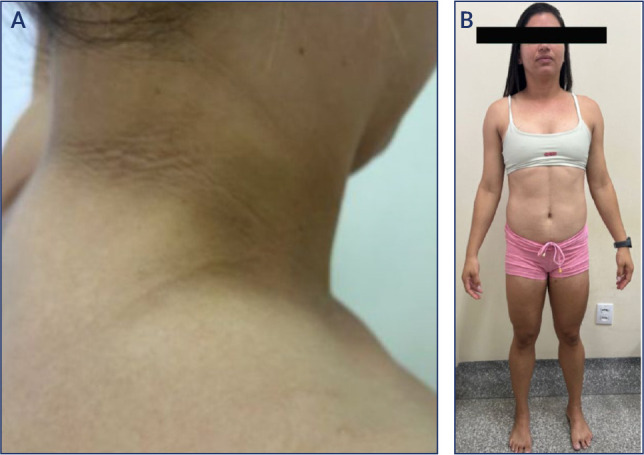
(**A and B**). Acanthosis nigricans in a woman with congenital partial lipodystrophy and severe hypertriglyceridemia, who exhibits acromegalic facies, truncal adiposity, muscle hypertrophy, polycystic ovary syndrome, liver steatosis, prediabetes, and hypertension. The diagnosis of congenital partial lipodystrophy led to intensified treatment, which improved the patient’s comorbidities and quality of life, and certainly prolonged her lifespan.

**Figure 2:**
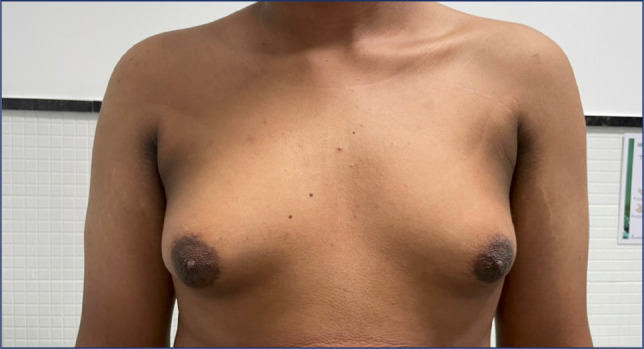
Axillary and cervical acanthosis nigricans and bilateral gynecomastia in a male patient with obesity and hypogonadotropic hypogonadism. Treatment of hypogonadism and obesity improved the patient’s acanthosis nigricans and probably reduced his cardiovascular risk.

**Figure 3:**
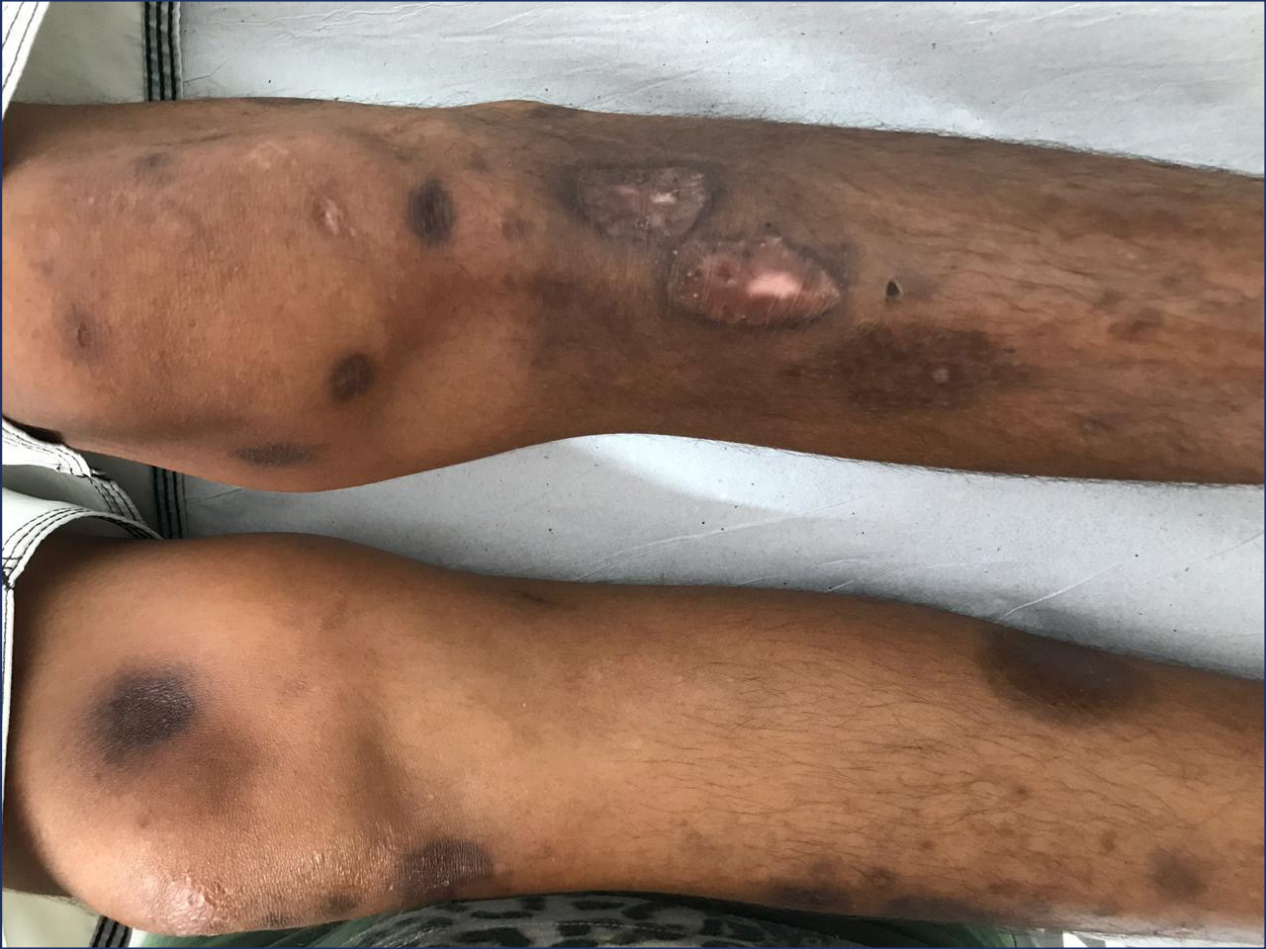
Diabetic dermopathy in a male patient with poorly controlled type 1 diabetes. Intensification of the insulin regimen led to an improvement of the lesions and, subsequently, better metabolic control, with benefits in terms of future complications.

**Figure 4:**
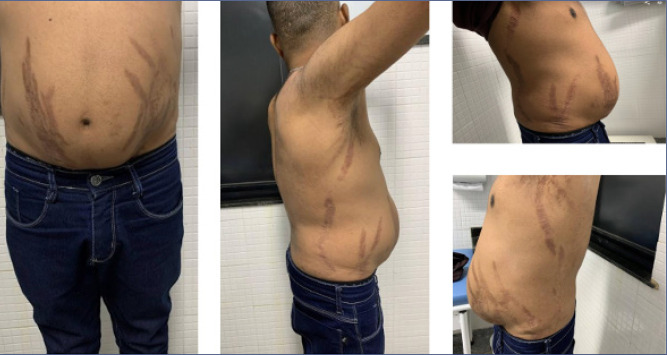
Broad, violaceous striae in a patient with adrenocorticotropic hormone-dependent Cushing’s syndrome due to ectopic adrenocorticotropic hormone secretion (thymic neuroendocrine tumor). At 18 months after tumor resection, the disease remained in remission.

**Figure 5:**
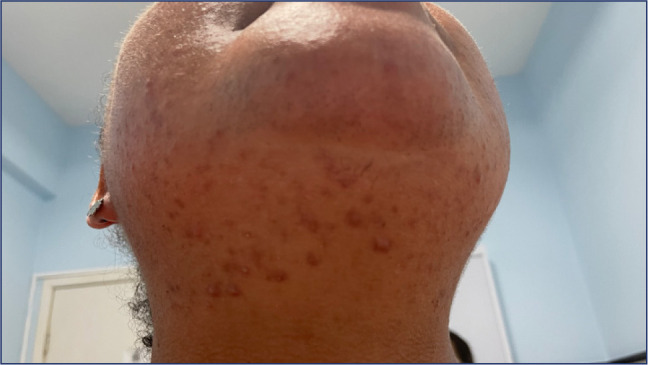
Hirsutism and folliculitis in a female patient with central obesity, diabetes, and hypertension due to Cushing’s disease. After pituitary surgery, the patient lost 17 kg, experiencing a drastic improvement in glycemic control and hypertension, along with a reduction in skin lesions.

Table 1 presents some of the skin findings observed in major hormonal imbalances. **Figures 1 to 5** display cases in which an accurate diagnosis led to life-saving therapeutic measures. Below, we describe important dermatoendocrine conditions related to the endocrine glands and the somatotropic system.

**Table 1:** Skin findings in major hormonal imbalances

Insulin resistance	Acanthosis nigricans, acrochordons, xanthelasma, eruptive xanthomas, androgenetic alopecia
Diabetes mellitus	Necrobiosis lipoidica diabeticorum, granuloma annulare, bullae, diabetic dermopathy, ulcers, infections
Hyperthyroidism	Thin skin, fine and downy hair, hyperhidrosis, facial flushing, palmar erythema, thyroid acropachy, jaundice, urticaria, onycholysis, alopecia areata
Hypothyroidism	Xerosis cutis, myxedema, pallor, carotenemia, vitiligo, alopecia areata, trichodystrophy, madarosis, frail nails, onycholysis
Hypercortisolism	Purple striae, facial plethora, acne, folliculitis, hirsutism, acanthosis nigricans, fungal infections, easy bruising
Hypocortisolism	Tanning, mucosal hyperpigmentation, vitiligo, loss of axillary and pubic hair
Hypoestrogenism	Dryness, itching, thinning, atrophy, wrinkling, sagging, urogenital atrophy, poor wound healing, hidradenitis suppurativa, reduced hair growth, increased facial hair growth
Hyperandrogenism	Androgenetic alopecia, sebaceous gland hypertrophy, thick skin, acne vulgaris, seborrhea, hirsutism, temporal baldness, acanthosis nigricans
Hyperparathyroidism	Pruritus, calcium deposition
Hypoparathyroidism	Hyperkeratotic and eczematous skin, brittle nails, sparse hair, mucocutaneous candidiasis
Growth hormone Deficiency	Thin and wrinkled skin, normal hydration and reduced sweating, decreased or normal sebum secretion, lower resistance and elasticity
Acromegaly	Thick skin, acanthosis nigricans, acrochordons, increased sebum and sweating, oily skin, acne

### Endocrine pancreas

Acanthosis nigricans is the most common dermatoendocrine manifestation of insulin resistance. It is characterized by symmetrical, hyperpigmented, velvety plaques with poorly defined borders, primarily affecting intertriginous areas (neck, axilla, elbow, and groin) (^24^), but it can also involve finger joints, particularly in Latinos, in whom it serves as an early marker of insulin resistance even in the absence of overweight or obesity (^25^). Insulin resistance is a crossroads of common diseases, including obesity, type 2 diabetes, hypertension, metabolic syndrome, dyslipidemia, and cancer (liver, pancreatic, endometrial, colorectal, and postmenopausal breast cancer); it is also involved in several diseases such as polycystic ovary syndrome, androgenetic alopecia, nonalcoholic steatohepatitis (^26^,^27^), and congenital (**Figures 1A and 1B**) or acquired lipodystrophies (^27^,^28^), among other serious conditions (**Figure 2**).

Another finding frequently associated with insulin resistance is the presence of acrochordons (or skin tags), which are benign, pedunculated skin growths commonly found in the neck, axillary, and groin regions (^25^,^29^). The presence of multiple acrochordons (eight or more) has been observed to be more sensitive than acanthosis nigricans in identifying patients with hyperinsulinemia (^30^). Hyperinsulinemia stimulates IGF-I receptors on fibroblasts’ cell surface (^31^), although other signaling pathways may participate in this process, including the epidermal growth factor cascade (^32^) and the leptin pathway (^33^). Multiple skin tags occur in about half of patients with acromegaly, suggesting GH dependence (^34^,^35^). However, we reported one isolated acrochordon in an individual with congenital isolated GHD who exhibited increased insulin sensitivity (^17^), emphasizing that it is the multiplicity, rather than the mere presence of skin tags, that suggests insulin resistance, as found in acromegaly. Other cutaneous conditions have also been reported in association with insulin resistance (and increased cardiovascular risk), such as ear lobe creases (by the involvement of structures beneath the skin), xanthelasma (associated with hypercholesterolemia), and eruptive xanthomas (associated with hyperchylomicronemia and severe hypertriglyceridemia) (^25^).

Another finding associated with insulin resistance and unfavorable cardiovascular outcomes is androgenetic alopecia, characterized by diffuse thinning of crown hair in women, with preservation of the frontal hairline in most, but not all cases (^36^). Androgenetic alopecia may be also associated with hyperandrogenic conditions other than insulin resistance in women, including polycystic ovaries, adrenocorticotropic hormone (ACTH)-dependent Cushing’s syndrome, and metabolic dysfunction associated with steatotic liver disease, which require preventive strategies with lifestyle modifications and pharmacological measures to avoid progression to liver cirrhosis and type 2 diabetes along with its costly complications (^37^,^38^). The Food and Drug Administration (FDA)-approved therapeutic options for androgenetic alopecia include topical minoxidil at 2% for women and 5% for men, oral finasteride (for men), hair restoration surgery, and low-level light therapy (^39^). Non-FDA-approved therapeutic options for men include oral dutasteride, while for women, options include cyproterone acetate, spironolactone, and topical estradiol (^39^).

Androgenetic alopecia must be distinguished from alopecia areata, an autoimmune disease that causes patchy hair loss on the scalp, face, and other parts of the body. It must also be differentiated from telogen effluvium, a type of non-scarring, often acute (but sometimes chronic) alopecia characterized by diffuse and excessive loss of resting or telogen hair (10 to 15% of scalp hair) following metabolic stress, hormonal changes, or medication use. Stressinduced alopecia involves multiple causes and is managed by a multidisciplinary team, with emphasis currently placed on the role of hormones from the hypothalamic-pituitary-adrenal axis (corticotropinreleasing hormone [CRH], ACTH, and cortisol) (^40^,^41^).

Skin manifestations can occur in most (if not all) patients with diabetes mellitus and are linked to some degree to glycemic control. They include necrobiosis lipoidica diabeticorum (erythematous, non-scaly papules in the pretibial area that evolve into dermal and epidermal atrophy and ulcers), granuloma annulare (quite similar to necrobiosis lipoidica diabeticorum but without epidermal atrophy), diabetic dermopathy (characterized by pigmented pretibial papules that begin as pink, 0.5 to 1 cm patches in the pretibial and lateral calf areas and progress to brown hyperpigmentation and surface atrophy with fine scaling) (**Figure 3**), ulcers, diabetic bullae, and thick skin. Several cutaneous infections - by candida, dermatophytes, and bacteria such as *Staphylococcus aureus*, beta-hemolytic streptococci (impetigo, folliculitis, furunculosis, carbuncles, ecthyma, cellulitis, and erysipelas), and *Pseudomonas aeruginosa* (malignant external otitis) - can also occur (^42^).

### Thyroid

Numerous interconnections exist between the skin and the thyrotropic axis, with cutaneous manifestations being conspicuous manifestations of thyroid hormone dysregulation (^6^). Hair follicles express thyrotropin-releasing hormone (TRH) and its receptor, as well as TSH and its receptor; the epidermis is also an extrapituitary source of TSH and a target for its effects (^43^,^44^). However, thyroid hormone action on the skin is primarily mediated by thyroid hormone receptors present in sebaceous glands, fibroblasts, smooth muscle cells, and vascular endothelial cells, as well as various cell types that constitute the hair follicle. Notably, T3 stimulates the growth of epidermal keratinocytes and dermal fibroblasts and inhibits the synthesis of hyaluronate (^6^).

Pretibial myxedema (or thyroid dermopathy) - characterized by well-demarcated pink or purple atrophic or transparent papules - is a well-known manifestation of Graves’ disease, with a 4:1 female-to-male ratio. Commonly coexisting with thyroid autoimmune ophthalmopathy, pretibial myxedema seems to arise from TSH receptor stimulation by substantially elevated antibodies in skin fibroblasts (^45^), most commonly confined to the pretibial area (^44^). Other cutaneous findings in hyperthyroidism include thin (yet not atrophic) skin, fine and downy hair (^46^,^47^), hyperhidrosis, facial flushing, and palmar erythema. More rare skin manifestations of hyperthyroidism include thyroid acropachy (digital swelling and nail clubbing associated with thyroid ophthalmopathy and dermopathy) (^48^), jaundice (likely due to an imbalance between hepatic oxygenation and blood flow reducing bile transport and cholestasis) (^49^), onycholysis or Plummer’s nails (distal separation of the nail plate from the nail bed), alopecia areata (hair loss without scarring, found in both Graves’ disease and Hashimoto’s thyroiditis) (^50^), chronic (^51^) or acute urticaria (due to hyperthyroidism or use of antithyroid agents) (^52^), and urticarial vasculitis (^53^).

Xerosis cutis (excessively dry and scaly skin) is the most common skin finding in hypothyroidism, whereas myxedema (thyroid dermopathy) is the most classic. The former results at least partially from a reduction of T3 binding to its receptor, causing hyperkeratosis (^54^), which occasionally takes on the appearance of ichthyosis (large fish-like thick scales that are adherent to the skin). Patients with myxedema have non-pitting edema in periorbital and pretibial regions (with abundant diffuse mucin within the dermal fenestrations as large amounts of glycosaminoglycans in the dermis), together with facial changes and sometimes macroglossia (^55^). Other skin manifestations of hypothyroidism include pallor (due to cutaneous vasoconstriction) and carotenemia (due to deficient conversion of carotene into vitamin A). The yellowish appearance of carotenemia can be mistaken for jaundice; one way to distinguish between the two is the involvement of the sclera, which is spared in carotenemia but not in jaundice.

Patients with autoimmune hypothyroidism (Hashimoto’s thyroiditis) may develop vitiligo, an autoimmune destruction of melanocytes and hypopigmentation (^56^). The most common hair manifestations of hypothyroidism are alopecia and trichodystrophy, which is characterized by dry, coarse, brittle, and slowgrowing hair that sheds easily (^6^). Alopecia develops due to an increased amount of hair in the telogen phase. Patients may also exhibit madarosis, a specific type of alopecia characterized by the loss of the lateral third of the eyebrows and the loss of eyelashes. After starting levothyroxine treatment, patients with hypothyroidism may initially experience worsening of alopecia as more hair sheds while follicles transition from the telogen to the anagen phase (^57^). The most common nail manifestations in hypothyroidism are increased fragility, slow growth, thinning, onycholysis, and brittleness (^58^).

### Adrenal

The skin is a barrier between the external and internal milieu and is, therefore, exposed to various external stressors, including UV radiation, injury, and foreign antigens, which can lead to an inflammatory response. Notably, the skin can synthesize glucocorticoids *de novo*. Recent research has revealed that these glucocorticoids - synthesized locally by keratinocytes mainly but also by melanocytes, dermal fibroblasts, and hair follicles - are likely to act in a paracrine or autocrine manner and have important physiological roles in local homeostasis, cellular development, and immune cell activation (^59^). Importantly, keratinocytes can also synthesize and activate cortisol (^60^). In fact, the skin expresses all elements regulating the activity of the hypothalamic-pituitaryadrenal axis, including CRH, urocortin, and proopiomelanocortin (POMC), along with their products ACTH, alpha-melanocyte-stimulating hormone (MSH), and beta-endorphin (^59^). The presence of the corresponding receptors on the same cells suggests that paracrine or autocrine mechanisms of action are involved (^61^). Recent evidence suggests that angiotensin II plays a role in cutaneous wound healing by promoting the differentiation of bone marrow-derived mesenchymal stem cells into keratinocytes (^62^). Blocking of the mineralocorticoid receptor alleviates glucocorticoid-induced skin atrophy in healthy human skin explants (^63^).

Since at least 1% of the population chronically uses glucocorticoids as anti-inflammatory or immunosuppressive agents, cutaneous manifestations of exogenous hypercortisolism are quite common; however, these manifestations can also reveal severe cases of endogenous hypercortisolism (^64^). These include purple striae (**Figure 4**), facial plethora, acne, folliculitis, hirsutism (**Figure 5**), acanthosis nigricans, fungal infections, and easy bruising. Fortunately, most cases resolve with the discontinuation of exogenous glucocorticoids or within 1 to 2 years after resolution of endogenous Cushing’s syndrome (^65^).

In primary adrenal insufficiency, the reduced cortisol production by damaged adrenal glands leads to increased production of CRH, POMC (an ACTH precursor), and MSH. Elevated MSH levels consequently trigger melanin synthesis by epidermal melanocytes, causing the tanning of the skin typical of Addison’s disease (^47^,^66^). This hyperpigmentation is most prominent in areas exposed to sun or trauma, such as scars and nevi, as well as the axillae, areolae, perineum, and palmar creases. Hyperpigmentation of mucosal surfaces may also occur and can be observed on the tongue and the inner surface of the lip, as well as in the buccal, gingival, and vaginal mucosa (^67^,^68^). Vitiligo, caused by autoimmune destruction of dermal melanocytes, occurs in 10 to 20% of individuals with autoimmune adrenalitis (^69^). Women with Addison’s disease may experience loss of axillary and pubic hair, which is caused by the loss of adrenal androgen production (^70^). This manifestation is also observed in secondary hypocortisolism (in the context of hypopituitarism), in which skin pigmentation is clearly lacking (^71^).

### Gonads

Estrogens contribute to increased skin thickness (through the production of collagen types I and III and transforming growth factor beta), moisture (by increased water retention in the stratum corneum and content of glycosaminoglycans, mucopolysaccharides and hyaluronic acid in the dermis), skin pigmentation (through estrogen receptors on melanocytes), and hair growth (by prolonging the anagen period through receptors on hair follicles that in turn express the enzymes 5-alpha reductase, aromatase, and 17-beta hydroxysteroid dehydrogenase, which are involved in estrogen synthesis) (^44^,^72^-^74^). Not surprisingly, pregnancy increases skin pigmentation and prolongs the anagen phase of hair growth. Additionally, estrogens and androgens stimulate sebum production (^75^).

Often overlooked, the cutaneous and mucosal manifestations of menopause are the most common dermatological findings associated with endocrine conditions and have a substantial negative impact on quality of life. They include dryness and itching, thinning and atrophy, wrinkling and sagging, poor wound healing, and reduced vascularity (^75^,^76^), along with an increased risk of other conditions such as hidradenitis suppurativa (due to relative androgen excess) and psoriasis (^77^). Hair symptoms of menopause include reduced hair growth and density on the scalp (including diffuse effluvium due to follicular rarefaction and female pattern androgenetic alopecia), disturbed hair quality and structure, and increased hair growth in facial areas (^77^,^78^). Estrogens also induce glycogen production by vaginal and cervical epithelial cells. The decline in estrogen at menopause results in urogenital atrophy and loss of vaginal epithelial cells (^79^), low levels of lactobacilli, fewer antimicrobial and anti-inflammatory agents, and increased susceptibility to mucosal lesions (^80^) and dyspareunia, further worsening quality of life. As in other dermatoendocrine conditions, treatment of the endocrine condition (hormone treatment) is expected to reverse the dermatological manifestations.

Progesterone can also influence hair follicle growth through central action (inhibitory effect on luteinizing hormone [LH] secretion, causing a decrease in thecal synthesis of androgens), as well as action on the hair follicle, decreasing the conversion of testosterone to dihydrotestosterone (through inhibition of 5-alpha reductase activity) (^81^). The primary role of progesterone is to counteract the effects of estrogen on the endometrium (^82^). It is unclear whether progesterone deficiency may contribute to the clinical manifestations of menopause-related relative hyperandrogenism or whether progesterone replacement may alleviate them.

The relative increase in androgens (testosterone and dihydrotestosterone) during menopause leads to miniaturization of hair follicles and decreased body and scalp hair, and may cause androgenetic alopecia (^83^), transforming small light vellus hairs in hormonesensitive areas on the face into darker terminal hair follicles, as well as causing hypertrophy of the sebaceous glands (^83^,^84^).

Outside the universal context of menopause, hypoestrogenism (of ovarian or central origin) or hyperandrogenism (of gonadal, adrenal, or exogenous origin) may present characteristics similar to those of menopause, often with greater severity. Androgenrelated disorders are most commonly due to increased sensitivity of the pilosebaceous unit to normal plasma androgen levels. Among the conditions associated with excess androgen, polycystic ovary syndrome is the most prevalent, affecting over one in ten women worldwide (^83^,^85^). Other causes of hyperandrogenism are congenital adrenal hyperplasia, ovarian and adrenal tumors, and drugs (anabolic steroids, progestogens, danazol) (^70^). Depending on the hyperandrogenism severity, the skin becomes thickened, rough and oily, with acne vulgaris and seborrhea, often accompanied by androgenetic alopecia, acanthosis nigricans, and hirsutism (increased androgen-dependent terminal hair growth on the lips, chin, chest, abdomen, linea alba, lower back, buttocks, inner thighs, and external genitalia). The most severe cases of hyperandrogenism are accompanied by virilization, which may include temporal baldness, voice deepening, muscle mass increase, feminine body contour loss, and clitoromegaly (^70^). In a retrospective analysis of 228 patients investigated for hyperandrogenism, 44.3% had hirsutism with elevated androgens and eumenorrhea, 27.6% had polycystic ovary syndrome (defined as hirsutism, elevated androgens, and oligomenorrhea), 22.4% had normal androgens with acne or alopecia and eumenorrhea, 3.1% had isolated low sex-hormone binging globulin (SHBG), 1.8% had nonclassical congenital adrenal hyperplasia, and 0.9% had ovarian tumors. In the study, the hirsutism score correlated positively with serum androstenedione, dehydroepiandrosterone sulfate, and salivary testosterone levels, and negatively with SHBG levels (^86^). Salivary testosterone had the highest correlation coefficient. These data illustrate the clinical and laboratory heterogeneity of hyperandrogenism (^87^).

### Parathyroid

Cutaneous manifestations of hyperparathyroidism are scarce and include pruritus and calcium deposition. In hypoparathyroidism, the skin is dry, scaly, hyperkeratotic, eczematous, and swollen. The nails become dull and brittle, developing transverse grooves. The hair becomes coarse and sparse (^70^). Mucocutaneous candidiasis may be observed in autoimmune hypoparathyroidism (^88^). Chronic hypoparathyroidism can also cause brittle nails and dry, scaly skin, in addition to its well-known manifestations of lethargy, weakness, fatigue, cataract, personality changes, and eventual permanent brain damage - all of which could be prevented by observing the skin (^89^). Skin inspection may aid in the diagnosis of calciphylaxis, a serious condition associated mainly with hyperparathyroidism secondary to chronic renal failure, but rarely occurring in patients with normal renal function, with a 2-year mortality rate of 50 to 80% due to sepsis. It occurs due to calcium deposition in arterioles, leading to ischemic ulceration of the overlying skin (^90^). Parathyroidectomy may be justified in some cases (^91^).

### Somatotropic system

As mentioned above, the separation between the internal and external environment through the skin was fundamental for the evolution of prevertebrates - whose growth occurs through extrapituitary circuits such as insulin, insulin similar growth, and local production of growth factors - into vertebrates - whose growth occurs through the somatotropic axis (*i.e.*, pituitary GH and circulating IGF-I) (^2^,^15^,^16^). As the skin covers the entire body, the somatotropic axis is expected to have a considerable influence on its structure and function. However, considering that the skin long preceded the appearance of vertebrae in animal evolution, the extrapituitary circuits, which preceded the somatotropic axis, may retain important actions on the skin in vertebrates and, consequently, in humans (^2^), as discussed below. All skin cell types express GH receptors, while keratinocytes and their stem cells also express the IGF-I receptor, enabling direct actions of GH or indirect actions involving the synthesis and modulation of IGF-I. This intricate process contributes to the homeostatic regulation of cell differentiation and proliferation within the skin tissue (^11^,^92^). In this sense, fibroblast-derived IGF-I is the main orchestrator of skin aging, integrating GH, estrogens, retinoids, and melatonin effects. The release of IGF-I is profoundly reduced in senescent fibroblasts (^93^). Insufficient IGF-I concentrations suppress cell growth, resulting in reduced ribosome numbers, suppressed protein translation, skin atrophy, and aging (^4^). Keratinocytes *per se* are unable to synthesize IGF-I; however, along with their stem cells, they express the IGF-I receptor, as stated previously. Therefore, keratinocytes, their stem cells, and other stem cells in the skin critically depend on the local IGF-I production by fibroblasts (^4^,^11^).

An analysis of cutaneous manifestations associated with conditions of GH excess or deficiency highlights the importance of the interrelationship between the somatotropic and integumentary systems. For example, individuals with acromegaly exhibit thickened skin, acanthosis nigricans, numerous pigmented acrochordons, and increased size and function of sweat and sebaceous glands, resulting in pronounced body odor, excessive sweating, oily skin, and acne (^94^-^96^).

The cutaneous consequences of GHD are difficult to clearly characterize, as they are often described in patients with acquired GHD who also have concomitant pituitary deficiencies that may not be adequately replaced (e.g., thyroid hormones, sex steroids, glucocorticoids) - all of which can greatly impact the skin. In this context, the skin of individuals with GHD is characterized by thinness, wrinkling, reduced sweating (^97^), and decreased sebum secretion (^98^-^100^). A cleaner model to assess the effect of GHD is represented by a cohort of individuals with severe isolated GHD caused by a homozygous mutation (c.57+1G→A) in the GHreleasing hormone (GHRH) receptor (GHRHR) gene (*GHRHR* OMIM n. 618157), described in the Brazilian city of Itabaianinha (*i.e.*, the Itabaianinha syndrome) (^2^,^15^-^20^,^101^,^102^). These individuals maintain consistently low levels of circulating pituitary GH and IGF-I throughout their lives (^102^), likely exhibiting compensatory extrapituitary circuits in important organs such as the skin. Due to the specific cause of GHD, these individuals have otherwise normal anterior pituitary function. They enjoy normal longevity (^103^) and quality of life (^104^) with high levels of physical activity (^105^) and abundant sun exposure through outdoor activities, which may influence skin health, photoaging, and the frequency of skin cancer (^17^).

These individuals with untreated isolated GHD exhibit wrinkled skin (^2^,^15^-^19^) and reduced sweating (^20^). The wrinkled skin reflects their lower skin resistance and elasticity (^2^), likely reflecting reduced content of collagen and elastin, which are crucial for the mechanical properties of the skin: collagen provides mechanical resistance to extension, while elastin allows deformation, providing a tailored mechanical response to accommodate body flexibility and movement and to prevent tears (^106^). The wrinkled skin in these individuals with isolated GHD seems to hold only cosmetic relevance, as they are very active in physical work and sports without easily tearing the skin (^2^,^15^-^19^). In contrast, sweating impairment has also been reported in patients with childhood-onset GHD (^107^), in men with adult-onset GHD (^108^), and in Laron’s dwarfism, which is caused by GH insensitivity, thus featuring IGF-I deficiency (^109^). Another important skin finding in the Itabaianinha cohort is that the frequency of photodamage and skin cancer does not differ from that in local controls. The frequency of skin cancer appears to be related to a significant familial component rather than isolated GHD *per se* (^2^,^17^). Their melanin content is normal in the forearm but lower in the buttocks, with hydration and sebum levels similar to those of controls (^2^). The normal melanin content in sun-exposed areas supports adequate photosensitivity, allowing prolonged sun exposure with few sequelae in terms of photoaging and cancer. Not surprisingly, these individuals have normal serum levels of 25-hydroxyvitamin D (^20^) and actually seem to have a healthier aging bone phenotype compared with controls (^110^). They present normal skin hydration (^2^), despite severely reduced sweating (^20^), as previously reported in hypopituitarism with GHD (^92^,^94^), but with normal skin sebum content (^2^). Accordingly, patients with acromegaly have increased sebum secretion (^94^). While in acromegaly the amount of sebum production appears to be related to GH and IGF-I levels, as well as disease duration and severity (^94^), the decrease in sebum secretion in GHD is only partially reversible by GH treatment (^100^), suggesting that other hormonal deficiencies may contribute to this reduction (^2^). Interestingly, the sebum content among the Itabaianinha individuals with isolated GHD is very similar to that of Turkish normal individuals, in whom sebum content was measured using the same methodology (^111^). Therefore, hydration and sebum production may also be maintained by extrapituitary circuits, without being severely impaired by the reduction in pituitary GH and circulating IGF-I levels (^2^).

## IMPLICATIONS FOR FUTURE RESEARCH

In this review, we have consolidated the concept that the skin, which is readily accessible through simple inspection, is a marker of endocrine diseases. The treatment of these endocrine diseases restores the skin and the general health of the affected individuals, potentially saving lives. This is particularly important in an era of digitized medical records, artificial intelligence, and online consultations. We encourage researchers to report other cases of cutaneous manifestations of endocrine diseases, with the same goal of saving lives and persuading journal editors of the importance of this effort.

In conclusion, through careful observation of the skin, we can diagnose and assess various endocrine diseases, improving patients’ quality of life and extending their life expectancy.

## Data Availability

datasets related to this article will be available upon request to the corresponding author.
